# Mitigation of Rumen Methane Emissions with Foliage and Pods of Tropical Trees

**DOI:** 10.3390/ani10050843

**Published:** 2020-05-13

**Authors:** Jorge Canul-Solis, María Campos-Navarrete, Angel Piñeiro-Vázquez, Fernando Casanova-Lugo, Marcos Barros-Rodríguez, Alfonso Chay-Canul, José Cárdenas-Medina, Luis Castillo-Sánchez

**Affiliations:** 1 Tecnológico Nacional de México/Instituto Tecnológico de Tizimín, Yucatán. Avenida Cupul km 2.5, Tizimín 97700, Mexico; jcanul31@gmail.com (J.C.-S.); majocn7@gmail.com (M.C.-N.); jose.cardenas@ittizimin.edu.mx (J.C.-M.); 2 Tecnológico Nacional de México/Instituto Tecnológico de Conkal, Conkal 97345, Mexico; pineiroiamc@gmail.com; 3 Tecnológico Nacional de México/Instituto Tecnológico de la Zona Maya, Othón P. Blanco 77960, Mexico; fkzanov@gmail.com; 4 Facultad de Ciencias Agropecuarias, Universidad Técnica de Ambato, Carretera Cevallos-Quero, Tungurahua 180350, Ecuador; ma.barros@uta.edu.ec; 5 División Académica de Ciencias Agropecuarias, Universidad Juárez Autónoma de Tabasco, Villahermosa 86280, Mexico; aljuch@hotmail.com

**Keywords:** climate change, ruminants, secondary metabolites, saponins, volatile fatty acids

## Abstract

**Simple Summary:**

Methane produced by enteric fermentation contributes to the emission of greenhouse gases (GHG) into the atmosphere. Methane is one of the GHG arising from anthropogenic activities with the greater contribution to global warming. This paper provides a brief introduction to the potential use of tropical foliage trees, pods, and secondary metabolites to reduce methane emissions from ruminant supply chains. A better knowledge of the available strategies for efficient foliage use in the tropics is essential in order to ensure increasing livestock production while preserving the environment. The mitigation of rumen methane production through the use of the foliage and metabolites of tropical trees represents an interesting challenge for scientists working in the field of ruminant nutrition.

**Abstract:**

Methane produced by enteric fermentation contributes to the emission of greenhouse gases (GHG) into the atmosphere. Methane is one of the GHG resulting from anthropogenic activities with the greater global warming contribution. Ruminant production systems contribute between 18% and 33% of methane emissions. Due to this, there has been growing interest in finding feed alternatives which may help to mitigate methane production in the rumen. The presence of a vast range of secondary metabolites in tropical trees (coumarins, phenols, tannins, and saponins, among others) may be a valuable alternative to manipulate rumen fermentation and partially defaunate the rumen, and thus reduce enteric methane production. Recent reports suggest that it is possible to decrease methane emissions in sheep by up to 27% by feeding them saponins from the tea leaves of *Camellia sinensis*; partial defaunation (54%) of the rumen has been achieved using saponins from *Sapindus saponaria*. The aim of this review was to collect, analyze, and interpret scientific information on the potential of tropical trees and their secondary metabolites to mitigate methane emissions from ruminants.

## 1. Introduction

Methane (CH_4_) gas is a byproduct of the anaerobic microbial fermentation of carbohydrates in the rumen [1,2], and it is one of the six greenhouse gases (GHG) included in the Kyoto Protocol, with a global warming potential 23 times that of Carbon dioxide (CO_2_) [3,4]. Among agricultural activities, ruminant production is one of the major sources of GHG emissions, contributing about 18% to 33% of the total CH_4_ emitted into the environment [4,5,6,7]. This is due to the fact that between 2% and 12% of the gross energy consumed by the ruminant is converted into CH_4_ during rumen fermentation [8]. Over recent years, there has been growing interest in predicting CH_4_ emissions from ruminant species in order to reduce emissions [9,10]. New strategies include the use of plant secondary metabolites [11,12].

Ruminant production systems in the tropics are characterized by grazing native and introduced grasses which present fluctuations in quantity and quality throughout the year [13]. The relatively low quality of tropical forages determines, to a large extent, an increasing fibrous material intake and, therefore, the production of rumen CH_4_ [14,15]. In this sense, tropical trees (TT) may contribute to an improvement in ruminants’ feeding due to their high nutritive value (136 to 325 g crude protein (CP/kg) dry matter (DM) and 50 to 60% apparent digestibility) [16]. Furthermore, TT contain a range of secondary metabolites [17,18], which could alter rumen fermentation [19,20], partially defaunate the rumen [21], and consequently reduce CH_4_ emissions [22,23].

The aim of this review was to collect, analyze, and interpret scientific information on the potential of using tropical trees and their secondary metabolites to mitigate CH_4_ emissions from ruminants.

## 2. Greenhouse Gases and Animal Production

Carbon dioxide (CO_2_), nitrous oxide (N_2_O), hydrofluorocarbons (HFCs), perfluorocarbons (PFCs), sulphur hexafluoride (SF_6_), and methane (CH_4_) are the main greenhouse gases (GHG) produced by global livestock [24]. The Intergovernmental Panel on Climate Change [3] reported for the period of 1970 to 2004 increases of 70% and 40% in the emission of CO_2_ and CH_4_, respectively. According to current data, the world human population has reached ≈ seven billion; however, it is expected to rise to nine billion by 2050 [25,26]. The projected population growth will drive up global demand for food and livestock production. In particular, it is estimated that meat consumption will increase from 229 to 465 million tons between the years 2000 and 2050, and the demand for dairy products will likely reach 1045 million tons [5]. As a result of the increased demand for animal-based protein, CH_4_ emissions are predicted to rise exponentially [27].

For example, studies conducted in Mexico showed that in 2015, CH_4_ emissions reached a magnitude of 70 567.60 Gg CO_2_e, with enteric fermentation making up 76% of the total CH_4_ released into the environment [28]. This was partly due to the growing livestock population reported for the period of 2006 to 2015 (33.5 million cattle, nine million goats, and nine million sheep) [29].

In 2015, García-Apaza et al. [30] forecast a linear growth rate of CH_4_ emissions deriving from the livestock sector in Bolivia. The aforementioned values were calculated following the Intergovernmental Panel on Climate Change (IPCC) recommendations [3], which in turn are based on estimates of cattle inventories. As a consequence, the estimate’s precision strongly depends on the availability and reliability of such information.

In Mexico, in order to establish the most appropriate strategies towards CH_4_ mitigation, it is necessary to develop precise emission factors with the purpose of having a reliable inventory of the magnitude of enteric CH_4_ emissions and a well-established livestock policy.

## 3. Overview of Methanogenesis in the Ruminants

Methane production by ruminants is a natural process which originates in the rumen during feed digestion [31]. In this process, several microorganism species known as methanogens convert feed such as proteins and starch into amino acids and sugars which are then fermented to become volatile fatty acids, while molecular hydrogen (H_2_) released during the production of acetate and butyrate in the rumen [32,33] and CO_2_ are reduced to CH_4_ [34].

The amount of methane produced in the rumen depends on the characteristics of the diet consumed by the animals [35,36]. By knowing the exact dry matter intake [37,38] and, consequently, the quantity of volatile fatty acids produced in the rumen, it is possible to calculate the total amount of methane that ruminants will emit [39]. Further studies on ruminal function and metabolic variables are needed in order to gain deeper insights into the effects of tropical plant foliage and secondary metabolites on livestock-derived GHG emissions.

## 4. Potential of Tropical Trees for the Feeding of Ruminants

A large diversity of tropical tree species could potentially be used to feed ruminants and improve livestock production [40,41,42]. The content of crude protein deriving from tropical tree foliage and fruit has a range of 136–325 g/kg dry matter (DM) and 79–429 g/kg DM, respectively, with a digestibility rate of 50–60% (Table 1) [20]. The productive performance (weight gain, milk yield) of ruminants is the best reflection of feed quality.

In the literature, many studies support this correlation. In Pelibuey lambs, for example, a moderate weight gain (90 g/head/day) has been observed after including 12% of *Acacia farnesiana* fruit in their diet [51]. Brown et al. [52] found that adding around 40% to 50% of *Acacia karroo* foliage in the Pedia goat diet based on *Setaria verticillata* leads to a higher DM, organic matter (OM), neutral detergent fibre (NDF), and acid detergent fibre (ADF) digestibility compared to the results obtained by including only 20%, 25%, and 30% of *A. karroo* foliage. Similarly, it has been shown that the use of 15% and 30% of *Gliricidia sepium* and *Enterolobium cyclocarpum* foliage, respectively, in the cross-heifer ration improves animal productivity due to their crude protein (CP), tannin, and saponin content [53].

In another study on bull diet, it was observed that replacing cotton seeds with *Morus alba* (0%, 5%, 10%, and 15% of the total ration) resulted in significant weight gain (554, 583, 565, 568 g/head/day, respectively) [54]. However, the substitution of milled sorghum with milled *E. cyclocarpum* fruits (0%, 12%, 24%, and 36% of the DM ration) had no significant effects on the productive performance of hair sheep [55].

Regarding the consumption rate, the incorporation of 45% of the ground fruits such as *Acacia pennatula* (group one) or *E. cyclocarpum* (group two) added to the commercial concentrated feed in the Pelibuey sheep ration significantly increased the consumption rate compared to group three fed only with commercial concentrate feed (1155, 1123 vs. 933 g DM/day, respectively)[56]. On the other hand, the addition of 0%, 20%, 30%, 40%, and 50% of the ground fruit of *E. cyclocarpum* in the ration of hair sheep significantly decreased the digestibility of DM in the treatment with the highest amount of fruit (50%). This result could be explained by a higher NDF intake despite similar DM intakes among the various treatments (73, 87, 88, 94 and 91 g/kg^0.75^/day) [57].

Lastly, Ansari, Mohammadabadi and Sari [58] found that adding *Albizzia lebbeck* in the humpback camel diet did not affect the digestibility of dry matter and NDF; similar results were observed for the conventional alfalfa diet.

## 5. Secondary Metabolites in Tropical Forage Trees

Trees are part of a complex set of interactions between plants, animals, and insects [59]. Given those interactions, trees have developed mechanisms of defense such as spikes, fibrous foliage, growth patterns, and the presence of secondary metabolites against herbivory, pathogens, pests, and defoliation [60]. Secondary metabolites, for example, are known to reduce the palatability and voluntary feed intake as well as the dry matter and protein digestibility of forages [61]. The most commonly present secondary metabolites in tropical trees are: tannins, alkaloids, cyanogenic glycosides, and saponins (Table 2).

## 6. Effect of Secondary Metabolites of Tropical Trees on Rumen Fermentation

Due to public concerns for the dramatic increase in the use of chemical compounds such as ionophores and antibiotics in the ruminant production industry, there has been growing interest in finding alternative feed additives [60]. In this regard, secondary metabolites represent a valuable and sustainable option as they may be used to manipulate rumen fermentation (i.e., alter the molar proportions of volatile fatty acids and reduce biohydrogenation of unsaturated fatty acids) [60].

Among secondary metabolites, tannins and especially saponins seem to be the most promising alternative feed additives [8,60]. Condensed tannins (CT) comprise a diverse group of polyphenols found in a large number of plant species in which they are responsible for bounding and precipitating proteins. While a low concentration of CT has a beneficial effect on nitrogen utilization due to the protection of proteins against microbial degradation in the rumen, a high concentration of CT has a detrimental effect on the intake, digestibility, and weight gain [63].

Saponins are found in many plant species and consist of bioorganic compounds classified as glycoside steroids, triterpenoids, and steroidal alkaloids. More specifically, they are defined as glycosides of high molecular weight, with one or more hydrophilic sugar chains (glucose, galactose, xylose, arabinose, ramnose, or glucuronic acid) combined with lipophilic aglycones which are either triterpene or steroid molecules. The aglycone moiety is also known as sapogenin [61,64].

Given their vast biological role as emulsifiers and detergents, as well as their pharmacological hemolytic [65] and antiprotozoal properties [17,66], saponins have recently been proposed as a means of manipulating rumen fermentation. For example, interactions between saponins and membrane-bound cholesterol lead to unsuitability, lysis, and death of the cell [59]. Additionally, in vivo and in vitro experiments using tropical trees such as *Sapindus saponaria*, *Pithecellobium saman, Tithonia diversifolia*, and *E. cyclocarpum* have highlighted the effects of saponins as defaunating agents and modifiers of rumen fermentation [21,49,67].

**Figure 1:**
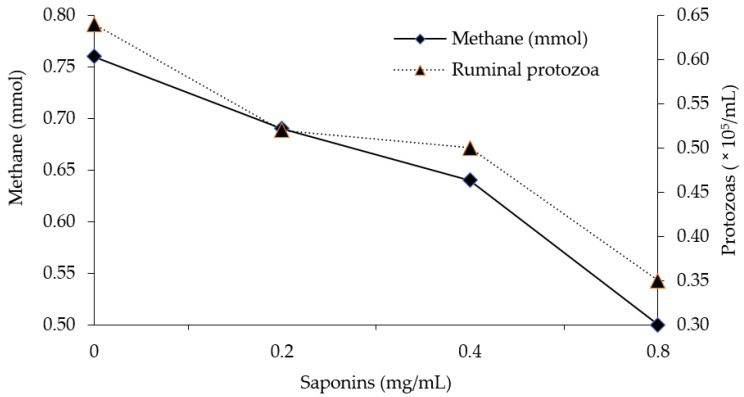
Effect of the inclusion of saponins on protozoa population and rumen methane (CH_4_) in vitro (Adapted from Hu et al., 2006 [70]).

Thus, the use of saponins as feed additives would highly benefit the environment and ruminant productivity as it has been shown that a reduction in the protozoa rumen decreases the total production of enteric CH_4_ while the use of dietary energy is increased (Figure 1) [53,68,69].

The potential of the forages and fruits of tropical trees for CH_4_ reduction, rumen defaunation, and changes in the molar proportions of volatile fatty acid (VFA) in the rumen has been demonstrated [20] (Table 3 and Table 4); however, conclusions are sometimes still contradictory.

Lila et al. [71] observed under in vitro conditions a linear decrease in the production of rumen CH_4_ as the level of saponins of *Yucca schidigera* in the ration was increased, with values ranging between 13.87, 10.96, 9.57, 7.25, and 5.82 mmol of CH_4_ for 0, 1.2, 1.8, 2.4, and 3.2 g/L of *Y. schidigera*, respectively. Another in vitro experiment using *Sapindus mukorossi* in diets based on wheat flour (80%) and wheat straw (20%) revealed a reduction of 22.68%, 11.48%, and 0% of methane in buffalo ruminal fluid when extracts of water, ethanol, and methanol were modified, respectively (Table 5) [72].

Conversely, studies on tropical plants such as *G. sepium* and *E. cyclocarpum*, and *Y. schidigera*, concluded that saponins did not reduce CH_4_ production under in vitro conditions [77], and no significant effects were reported on ruminal methane production under in vivo conditions when Pelibuey sheep were fed with *P. purpureum* and supplemented with increasing levels of *Yucca schidigera* saponins (0, 1.5, 3.0, and 4.5 g/day) [78]. Akanmu et al. [79] reported under in vitro conditions that the addition of 50 mg/kg of *Moringa oleifera* and *Tithonia diversifolia* extracts to a forage-based diet reduced CH_4_ production without adverse effects on feed digestibility.

Pen et al. [80] found that using 2 to 6 mL/L liquid extract of *Y. schidigera* and *Quillaja saponaria* induced a partial defaunation of the rumen, a change in the proportion of propionate, a reduction of the ratio of acetate to propionate, and a decrease in CH_4_ production from 32% to 42%. Similar results have been reported by Bekele et al. [73], who observed a reduction of CH_4_ of 13% and 34% when adopting *Acacia angustisima* and *Sesbania sesban,* respectively. A reduction in CH_4_ emissions has also been recorded with the use of saponins from *Y. schidigera* and *Q. saponaria* as a result of the negative effect on the digestibility of NDF [81], mainly caused by the reduced activity of rumen bacteria during NDF fermentation [59]. Furthermore, a decrease of 10% and 27% of CH_4_ production was documented in the rumen of goats and sheep, respectively, when saponins from tea leaves were added to their diet [23,70,82].

The daily use of 880 and 2640 mg of saponin from powdered *Y. schidigera* in bulls increased the proportion of propionate (2.8 y 3.0 mmol) compared to a diet without saponins, which in turn leads to a lower CH_4_ production. Additionally, it has been demonstrated that the use of 880 mg of saponins reduces protozoa population by 42%, while at a higher dose (2640 mg) no further effects on defaunation were recorded [83]. Likewise, it was recorded that by introducing 187 g DM of leaves of *E. cyclocarpum* in the ration (14.96 g of saponins) of sheep fed barley silage and concentrate (60:40), it was possible to diminish the protozoa population in the rumen by 25% [21].

CH_4_ emissions can be reduced up to 70% when feeding goats (8 kg live weight) with *G. sepium* as a basal ration (214 g DM/day) compared to a control ration [84]. However, the use of 45% of *Acacia pennatula* and *E. cyclocarpum* in sheep’s diets did not result in lower CH_4_ emissions (237 and 219 vs. 196 kJ/mol of the control group) [56].

Experiments on dairy cows recorded no reduction in rumen CH_4_ when saponins from *Y. schidigera* and *Q. saponaria* were added in doses of 10 g/kg of DM [81]. Probably, this is related to the type of saponins since previous studies reported a significant effect on CH_4_ production using similar doses of another type of saponin [81]. Several authors suggested that the lack of long-term effects of saponins is likely due to the adaptation of rumen microorganisms to these metabolites [85,86]. This finding is supported by the results obtained in steers fed a basal ration (corn and maize silage) with the addition of 1.5%, 1.5%, and 0.5% of saponins from *Y. schidigera*, *Q. Saponaria*, and *Camelia sinensis,* respectively, which indicate that those levels and types of saponins did not affect the daily emission of CH_4_ [87].

However, the use of *Leucaena leucocephala* caused a reduction in the daily CH_4_ emission of 11–31.56% when the legume was increased from 22% to 44% of the total DM intake [42,75,88]. Table 5 and Table 6 show evidence of the effects of saponins from tropical trees on rumen fermentation, rumen microbial population, and CH_4_ emissions. Diversity of the results are reported in the literature regarding the effect of tropical tree metabolites on ruminal microorganisms and methane emission. Studies are still needed to better understand the action of these compounds in ruminal physiology.

## 7. Conclusions

This paper shows that the use of foliage and fruits from tropical trees as feed for ruminants represents a valuable and sustainable alternative in the developing countries of Latin America, particularly during those seasons characterized by lower forage quality and availability. The presence of secondary metabolites in tropical forage trees, especially saponins and tannins, may be used to manipulate rumen fermentation, partially defaunate the rumen, and, consequently, reduce the emission of enteric CH_4_ into the environment.

## Figures and Tables

**Table 1:** Chemical composition (g/kg of dry matter) of foliage, fruits, and leaves of forage trees.

Species	Fraction	OM	CP	NDF	ADF	References
*Acacia pennatula*	Foliage	929	125	590	358	[41]
*Cratylia argentea*	Foliage	-	273	587		[43]
*Erithryna berteroana*	Foliage	901	243	-	-	[44]
*Gliricidia sepium*	Foliage	894	238	385	247	[45]
*Guazuma ulmifolia*	Foliage	862	104	425	295	[46]
*Guazuma ulmifolia*	Foliage	-	110	520	344	[46]
*Hibiscus rosasinensis*	Foliage	-	266	367	223	[41]
*Leucaena leucocephala*	Foliage	898	201	275	191	[41]
*Leucaena leucocephala*	Foliage	-	245	452	255	[41]
*Morus alba*	Foliage	-	176	260	228	[47]
*Trichantera gigantea*	Foliage	-	199	407	339	[48]
*Acalipha villosa*	Foliage	899	162	361	291	[46]
*Ampelocissus erduendbergiana*	Foliage	934	157	494	332	[46]
*Brosimum alicastrum*	Foliage	-	142	375	260	[41]
*Crecopia obstusifolia*	Foliage	896	165	394	271	[46]
*Dalbergia glabra*	Foliage	941	187	629	415	[46]
*Galactia multiflora*	Foliage	925	137	409	232	[46]
*Guazuma ulmifolia*	Foliage	919	137	451	288	[46]
*Piscidia piscipula*	Foliage	905	126	500	346	[46]
*Psichotria nervosa*	Foliage	889	165	326	193	[46]
*Spondias mombim*	Foliage	892	148	283	197	[46]
*Tropis racemosa*	Foliage	878	130	345	297	[46]
*Acacia pennatula*	Fruits	955	85	720	487	[41]
*Enterolobium cyclocarpum*	Fruits	907	109	251	-	[49]
*Enterolobium cyclocarpum*	Fruits	966	164	339	221	[41]
*Guazuma ulmifolia*	Fruits	947	58	461	354	[41]
*Leucaena leucocephala*	Fruits	942	186	519	370	[41]
*Pithecellobium saman*	Fruits	920	147	291	-	[49]
*Enterolobium cyclocarpum*	Leaves	-	204	640	382	[50]
*Gliricidia sepium*	Leaves	-	195	526	299	[50]
*Leucaena leucocephala*	Leaves	-	216	687	412	[50]
*Moringa oleifera*	Leaves	-	254	632	411	[50]

CP: crude protein; OM: organic matter; NDF: neutral detergent fiber; ADF: acid detergent fiber.

**Table 2:** Concentration of the main secondary metabolites in foliage of tropical trees (g/kg DM).

Species	Fraction	TF	CT	SAP	References
*Acacia pennatula*	Foliage	29.0	40.0	-	[41]
*Albizia lebbeck*	Foliage	9.4	5.3	-	[62]
*Enterolobium cyclocarpum*	Foliage	1.4	1.5	8.0	[21]
*Erithrina variegata*	Foliage	2.2	0.2	-	[62]
*Gliricidia sepium*	Foliage	3.0	-	-	Laboratory *
*Leucaena leucocephala*	Foliage	5.0	1.8	-	[62]
*Moringa oleifera*	Foliage	4.0	2.9	-	[62]
*Enterolobium cyclocarpum*	Pods	-	52	19.0	Laboratory *
*Sapindus saponaria*	Pods	-	32	120.0	[49]

TF: total phenols; CT: condensed tannins; SAP: saponins; - without information; * laboratory analysis of experimental samples.

**Table 3:** Potential of foliage of tropical trees for methane (CH_4_) mitigation, rumen defaunation, and changes in the molar proportions of volatile fatty acids in vitro.

Species	CH_4_	CH_4_/Total Gas	Protozoa	VFA l/100 moL)			Reference
	(mL)	(*v:v*)	(10^4^/mL)	Ac	Pr	Bu	
*Pennisetum purpureum*	6.53	0.184	-	-	-	-	[20]
*Sesbania sesban* 10865	0.75	0.068	3.01	68	20	9	
*Samanea saman*	1.14	0.052	2.39	63	25	9	
*Acacia angustissima* 459	1.25	0.075	4.01	69	20	8	
*Acacia nilotica*	2.2	0.064	3.25	72	16	9	
*Leucaena leucocephala*	5.57	0.112	2.82	73	20	6	
*Sasbania sesban* 15019	6.56	0.144	3.77	70	20	7	
*Gliricidia sepium*	7.33	0.147	2.15	70	21	7	
*Moringa stenopetala*	7.72	0.15	2.72	71	20	7	

Ac: acetate; Pr: propionate; Bu: butyrate; CH_4_: methane; VFA: volatile fatty acids.

**Table 4:** Potential of foliage and seeds of tropical trees for methane (CH_4_) mitigation, rumen defaunation, and changes in the molar proportions of volatile fatty acids in vitro.

Species	CH_4_	CH_4_/total gas	Protozoa	VFA (moL/100 moL)			Reference
	(mL)	(*v*:*v*)	(10^4^/mL)	Ac	Pr	Bu	
*Pennisetum purpureum*	6.53	0.184	-	-	-	-	[20]
*Sapindus saponaria*	5.14	0.12	1.86	65	25	8	
*Leucaena leucocephala*	7.32	0.133	3.68	66	24	7	
*Albizia lebbeck*	7.95	0.137	0.62	64	23	10	
*bracteolate*	10.68	0.163	2.72	67	22	9	
*Enterolobium cyclocarpum*	12.71	0.175	2.1	63	27	9	
*Albizia saman*	16.01	0.205	5.16	69	21	8	

Ac: acetate; Pr: propionate; Bu: butyrate; CH_4_: methane; VFA: volatile fatty acids.

**Table 5:** Effect of metabolites from tropical trees on molar proportions of volatile fatty acids and CH_4_ production in the rumen.

Diet/Conditions and Quantity of Substrate	Source of Metabolites	Dose	Molar Proportion			CH_4_	References
			Acetate	Propionate	Butyrate	mmoL/day	
RUSITEC (14 g/day of mix grass: legume, 80: 20 in fermenters).	*Samanea saman 14884*	ND	63	27	7	3.61	[73]
	*Acacia angustissima 459*	ND	64	26	7	2.02	
	*Sesbania sesban 10865*	ND	63	28	7	1.55	
Basal diet	Sheep fed with concentrates	0:3	73	19	7	1.85	[74]
*B. brizantha: Cratylia argentea*		1:3	72	21	7	1.81	
		2:1	68	23	7	1.73	
Basal diet	Sheep fed with concentrates plus *S. saponaria* (7.71 g crude saponin/lamb/day) in each proportion	0:3	72	21	6	1.63	
*Cratylia argentea: B. brizantha*		1:3	70	23	6	1.68	
		2:1	69	23	7	1.64	
Isoenergetic and isoproteic balanced diets	*Neomillspaughia emargiata* *Tabernaemontana amygdalifolia* *Caesalpinia gaumeri* *Piscidia piscipula* *Leucaena leucocephala* *Havardia albicans*	1:3	61	25.8	13.92	1.73	[75]
Water flour (80%) Water straw (20%)	*Sapindus mukurossi* Water extract	20 g/100 mL of solvent	53.12	34.20	12.67	22.68	[72]
	Control	0	60.26	21.71	17.97		
	*Sapindus mukurossi* Methanol extract	20 g/100 mL of solvent	61.21	27.24	11.56	0	
	Control	0	61.48	27.4	11.21		
	*Sapindus mukurossi* Ethanol extract	20 g/100 mL of solvent	60.60	29.11	10.29	11.48	
	Control	0	61.10	29.92	8.97		
	Control	0	3.92	0.94	0.30	2.35	
HFD 80:20	*Myristica fragrans*	1 mL extract/100 mL	2.90	0.76	0.31	1.97	[76]
	Control	0	4.09	1.13	0.38	2.57	
LFD 20:80	*Myristica fragrans*	1 mL extract/100 mL	3.06	0.96	0.41	2.01	

RUSITEC: Ruminal simulation technique system; CH_4_: methane; ND: not determinate; HFD: high fiber diet; LFD: low fiber diet.

**Table 6:** Effect of metabolites of foliage of tropical trees on the rumen microbial population and CH_4_ reduction.

Species	Method	Treatments	Protozoa	Bacteria	Metanogens	References
			CFU/mL			
Basal diet	Sheep fed with concentrate	00:03	138	2930	452	[74]
*B. brizantha: Cratylia argente*		01:02	207	2530	484	
		02:01	154	2510	517	
Basal diet	Sheep fed with concentrate plus *S. saponaria* (7.71 g crude saponins/lamb/day) in each proportion	00:03	50	3530	493	
*Cratylia argentea: B. brizantha*		01:02	71	4010	697	
		02:01	91	4180	703	
Control	RUSITEC	0	6.3	3500	220	[49]
*Sapindus saponaria* (100 mg fruits/g diet)	120 mg saponins/g fruit	12	2.9	3300	210	
*Enterolobium cyclocarpum* (200 mg fruits/g diet)	19 mg saponins/g fruit	3.8	9.7	3300	210	
*Pithecellobium saman* (200 mg fruit/g diet)	17 mg saponins/g fruit	3.4	9.7	3400	230	

RUSITEC: Ruminal simulation technique system; CFU: colony forming units. Protozoa numbers × 10^3^; Bacteria and metanogen numbers × 10^6^; Diet: grass hay (620, 555, 498, 494), *Arachis pintoi* (248, 222, 194, 195), barley straw (120, 112, 100, 100), and urea (12, 11, 8, 11). Control diet (first value) and (second, third, and fourth value) represents inclusion levels g/kg DM diet ingredients in each tropical fruit tree.
